# Baohuoside I Combated *Cryptocaryon irritans* via Dual Targeting of Parasite Apoptosis and Host Defense Enhancement

**DOI:** 10.3390/antiox15030396

**Published:** 2026-03-21

**Authors:** Yan Lin, Li Huang, Yuan Yuan, Zhenyu Lin, Lei Huang, Tianxing Lin, Anqi Lin, Yuqi Zhu, Shoujie Jiang, Ying Huang, Yuqian Zheng, Rongjing Cai, Chengzhen Gu

**Affiliations:** 1 College of Bee Science and Biomedicine, Fujian Agriculture and Forestry University, Fuzhou 350002, China; 2 College of Marine Sciences, Fujian Agriculture and Forestry University, Fuzhou 350002, China; 3 College of Life Sciences, Fujian Agriculture and Forestry University, Fuzhou 350002, China

**Keywords:** *Cryptocaryon irritans*, baohuoside I, *Epimedium brevicornu*, mechanism, antiparasite

## Abstract

*Cryptocaryon irritans* Brown, 1951, a ciliated protozoan, is the pathogen of cryptocaryoniasis (white spot disease) in marine fish, causing substantial morbidity and mortality, particularly in tropical and subtropical regions. This is the first study to investigate the antiparasitic activity of baohuoside I, a natural flavonoid isolated from *Epimedium brevicornu* Maxim., against *C. irritans*. In vitro exposure to baohuoside I suppressed theront viability and tomont hatching in a dose- and time-dependent manner, inducing an apoptosis-like death in both stages, characterized by ciliary detachment, mitochondrial disruption, nuclear condensation, and extensive vacuolization, as evidenced by transmission electron microscopy and Annexin V-FITC/PI staining. Further studies demonstrated that baohuoside I elevated the intracellular Ca^2+^ and reactive oxygen species levels in tomonts, indicating Ca^2+^ overload and oxidative stress. Transcriptomic analysis of infected *Larimichthys crocea* skin revealed that baohuoside I upregulated immune-related genes while downregulating pro-inflammatory genes, concurrently enhancing host serum acid phosphatase activity and mitigating oxidative stress in enzyme activity assays. In vivo trials showed that oral administration of baohuoside I reduced trophont attachment and improved fish survival. It did not exhibit hemolytic activity at concentrations effective against the parasites. Collectively, these findings elucidate a multi-target mechanism of baohuoside I, highlighting its potential as an eco-friendly therapeutic agent for cryptocaryoniasis control in marine aquaculture.

## 1. Introduction

*Cryptocaryon irritans* Brown, 1951 is a pathogenic ciliate parasite that predominantly parasitizes the body surface, fins and gills of marine teleost and can infect a broad range of cultured species in tropical and subtropical regions, causing cryptocaryoniasis (white spot disease) and substantial mortality in aquaculture species such as *Sparus aurata*, *Dentex dentex*, *Larimichthys crocea*, *Seriola dumerili*, and *Trachinotus ovatus* [1]. *C. irritans* feeds on host body fluids, tissue fragments and cells, and the infected fish ultimately die from respiratory distress, osmotic imbalance, or secondary bacterial infections [2]. The life cycle of *C. irritans* comprises four stages—theronts, trophonts, protomonts, and tomonts. Theronts are highly infectious and disseminate through water flow, actively seeking hosts; following infection, trophonts extract nutrients from the host, detach, and develop into swimming protomonts that settle on the tank bottom and mature into tomonts. Tomonts proliferate and release theronts to initiate another infection cycle [3].

Current prevention and control of cryptocaryoniasis rely largely on physical methods and chemical methods. Physical methods, such as freshwater immersion, heating, ultraviolet irradiation, and ozone, can only reduce disease incidence in small systems and are impractical for large-scale or open culturing operations. Chemical methods include immersion or oral administration of chemical drugs, such as formalin, sodium hypochlorite, copper sulfate, antibiotics, etc. Although these drugs have therapeutic effects, the extensive use may leave chemical residues, promote resistance, and raise environmental concerns. Importantly, many agents predominantly kill free-living stages of *C. irritans* (tomonts or theronts) and exhibit limited efficacy against established trophonts [1]. Therefore, conventional physical and chemical measures can only delay the progression of disease but cannot completely cure it. Vaccination has been proposed as a preventive strategy; however, field protection remains suboptimal, and parasite immune evasion mechanisms continue to constrain vaccine effectiveness [4,5,6,7]. Hence, there is an urgent need for sustainable and multi-pronged interventions.

Natural products have attracted attention as sources of antiparasitic agents, yet practical application is hindered by limited supply and low yields. *Epimedium brevicornu* Maxim., a perennial herb in Berberidaceae with a long history in traditional Chinese medicine (TCM) to “reinforce kidney yang (a fundamental concept in TCM referring to the warming and activating energy of the body), strengthen muscles and bones”, offers abundant and relatively stable phytochemicals, including flavonoids, polysaccharides, essential oils, phytosterols, phenolic acids, and alkaloids, with pharmacological functions such as immunity enhancement, anti-cancer, anti-aging, cardioprotection, and learning and memory enhancement [8]. Among them, flavonoids are the major constituents, mainly involving baohuoside I/II, epimedin A/B/C, quercitrin, sagittatoside A/B, hyperoside, icariside I, icariin and astragalin, demonstrating broad bioactivities, including inhibition of malignancies through promotion of ferroptosis, modulation of the tumor microenvironment, enhancement of immunity, and exacerbation of mitophagy [8,9,10,11,12,13,14,15,16].

In this study, a flavonoid compound baohuoside I was purified and identified from *E. brevicornu*. We evaluated the antiparasitic potential of baohuoside I against *C. irritans*, focusing on its effects on theronts and tomonts in vitro, assessing protective efficacy in the susceptible large yellow croaker (*Larimichthys crocea*), and investigating potential mechanisms by examining parasite apoptosis, intracellular Ca^2+^ levels, oxidative stress, and gene expression to illuminate the mode of action. By exploring the preventive potential of baohuoside I, this study provides an environmentally friendly strategy for cryptocaryoniasis control and broadens the medicinal value of *E. brevicornu* for sustainable aquaculture.

## 2. Materials and Methods

### 2.1. Isolation of Baohuoside I from E. brevicornu

*E*. *brevicornu* were collected from Longnan, Gansu Province, P.R. China, and authenticated by Chengzhen Gu. The voucher specimens were deposited in the herbarium in the College of Bee Science and Biomedicine, Fujian Agriculture and Forestry University, with deposition numbers of Eb-202207. Dried *E*. *brevicornu* leaves was finely ground to a uniform powder (1.5 kg) and extracted with 95% ethanol for 24 h at room temperature. The filtrate was collected and the powder was re-extracted with 95% ethanol for another 24 h. This extraction process was repeated three times. The combined extracts were concentrated under reduced pressure using a rotary evaporator (N-1100S-W, TOKYO RIKAKIKAI, Tokyo, Japan) to yield 366.5 g of crude ethanol extract, which was redissolved in distilled water and partitioned successively with petroleum ether and ethyl acetate. The resultant ethyl acetate fraction was dissolved in methanol and subjected to chromatography on microporous resin HP-20SS (Mitsubishi, Tokyo, Japan) with a gradient methanol elution at concentrations of 10%, 20%, 30%, 40%, 50%, 60%, 70%, 80%, 90% and 100%. Each elution step used 1.5 L of methanol, and the corresponding fractions were collected. The resulting fractions were analyzed by thin-layer chromatography (TLC) and visualized by spraying the plates with an ethanol-sulphuric acid reagent. Fractions displaying the same TLC profiles were combined to yield eight fractions (Fr. 1–8). Fr. 1 was further chromatographed on MCI-gel CHP20P (Mitsubishi, Tokyo, Japan) and eluted with methanal from 10% to 100%. Eluents from each gradient were collected and analyzed by TLC as above. Fractions with identical chromatograms were pooled to yield four fractions (Fr. A–D). Fr. D was chromatographed on MCI-gel CHP20P and eluted with methanal from 20% to 70%. Eluents were collected, analyzed by TLC, and combined to yield four fractions (Fr. d1–d4). Fr. d2 was purified with MCI-gel CHP20P, filtered and precipitated to obtain a yellow powder (23.6 mg), designated as EFrd2.

### 2.2. Preparation of Fish and Parasites

A total of 300 large yellow croakers (*L. crocea*, 20 ± 5 g) were purchased from the standardized Ningde Fufa Fisheries Co., Ltd. (Ningde, Fujian, China). The fish were offspring of the farm’s own breeding stock and had no history of *C. irritans* infection or other parasitic diseases. Fish were examined for health status by inspecting the skin surface and gills under a microscope to confirm they were parasite-free. Prior to the experiment, fish were quarantined for 14 days with observation of behavior, feeding, and external health status; no signs of disease were detected. The fish had not been vaccinated against any fish pathogens. They were maintained in a 500 L recirculation seawater system and fed twice daily (10:00 and 20:00) at a rate of 3% body weight. Fish were acclimated to laboratory conditions (circulating seawater at 18 ± 2 °C, salinity 25 ± 0.5 ppt, pH 8.0 ± 0.5, dissolved oxygen 7.2 ± 1 mg/L) for 7 days before subsequent experiments. Healthy individuals of uniform size were randomly assigned to experimental groups to minimize potential differences in immune status.

*C. irritans* propagation was carried out according to previous studies [17,18]. Briefly, *C. irritans* tomonts were collected from the seawater in Ningde Fufa Fisheries where *L. crocea* was suffering from cryptocaryoniasis. Tomonts were washed with sterilized seawater and incubated in 25–27 °C to hatch the theronts. Theronts (8000 theronts/fish) were inoculated to *L. crocea* kept in a 50 L tank to propagate the next generation of *C. irritans*. Tomonts and theronts newly developed within 3 h were collected and used for the experiments. The concentrations of tomonts and theronts were determined by pipetting 100 μL into a well of a 96-well plate and counting under an inverted microscope (×20 magnification, Nikon eEclipse Ti, Tokyo, Japan).

### 2.3. In Vitro Antiparasitic Assay Against Theronts and Tomonts

For the test against theronts, baohuoside I was dissolved in 1% methanol and double-diluted with seawater to obtain a series of concentrations. A volume of 1 μL of baohuoside I solution was incubated with *C. irritans* theronts (100 individuals) in each well of the 96-well plate at 27 °C. Theronts were observed under an inverted microscope (×20 magnification, Olympus, Tokyo, Japan) at 30 min intervals to monitor cessation of movement, cell rupture, or deformation. Ten microliters of 4% paraformaldehyde was added to each well to terminate the reaction and fix theronts, and the total number of theronts was counted. The mortality was calculated as mortality (%) = (number of dead parasites at a given time point/total number of parasites) × 100%. The morphology of theronts was examined under an inverted microscope (Nikon eEclipse Ti, Tokyo, Japan), and the internal structures were observed with a transmission electron microscope (TEM, Hitachi, Tokyo, Japan). Each concentration was assayed in triplicate, and the experiment was independently repeated three times.

For the test against tomonts, 10 μL of baohuoside I was incubated with *C. irritans* tomonts (20 individuals) in each well of the 24-well plate for 6 h. The solution in each well was then replaced with aerated fresh seawater and incubated at 28 °C until the tomonts in the control group reached the theront stage. Parasites that lost internal motility, exhibited abnormal cell division, or could not produce theronts were considered dead. The morphology of tomonts was observed under an inverted microscope (Nikon eEclipse Ti, Tokyo, Japan), and the internal structures were examined with a transmission electron microscope (TEM, Hitachi, Tokyo, Japan). Each concentration was assayed in triplicate, and the experiment was independently repeated three times.

### 2.4. Apoptosis Detection

The apoptosis of *C. irritans* theronts and tomonts was detected using the Annexin V-FITC Apoptosis Detection Kit (C1062M, Beyotime, Shanghai, China) according to the manufacturer’s instructions. One hundred *C. irritans* theronts and tomonts were exposed to 40 μL of baohuoside I solution for 30 min and 12 h, respectively. After being washed twice, theronts and tomonts were then incubated in 100 μL of 1× Binding Buffer containing 2.5 μL of Annexin V-FITC and 2.5 μL of PI Staining Solution for 10 min. Apoptosis and necrosis of theronts and tomonts were observed by a laser scanning confocal microscope (Leica, Wetzlar, Germany) with excitation at 490 nm and emission at 525 nm.

### 2.5. Cytosolic Calcium Measurement

The concentration of cytosolic Ca^2+^ in tomonts was measured using the Fluo-4 Calcium Assay Kit (S1061M, Beyotime, Shanghai, China) according to the manufacturer’s instructions. Briefly, 100 individuals of *C. irritans* tomonts were exposed to 40 μL of baohuoside I solution (0, 10, 20 μg/mL) for 12 h. After being washed twice, tomonts were incubated with 250 μL of Fluo-4 AM at 37 °C for 30 min. Changes in intracellular Ca^2+^ concentration were observed under a fluorescence microscope (Nikon eEclipse Ti, Tokyo, Japan) with excitation at 490 nm and emission at 525 nm.

### 2.6. Reactive Oxygen Species (ROS) Detection

The production of ROS by tomonts was detected using the ROS Assay Kit (S0033M, Beyotime, Shanghai, China) according to the manufacturer’s instructions. Briefly, 100 individuals of *C. irritans* tomonts were exposed to 40 μL of baohuoside I solution (0, 10, 20 μg/mL) for 12 h. After being washed twice, tomonts were incubated with DCFH-DA (10 μM) at 37 °C for 20 min. ROS production was observed under a laser scanning confocal microscope (Leica, Wetzlar, Germany) with excitation at 488 nm and emission at 525 nm.

### 2.7. In Vivo Tests on Fish Challenged with C. irritans

Baohuoside I was dissolved in 1% methanol and diluted with deionized water. The baohuoside I solution was sprayed evenly onto the surface of the basal feed at a ratio of 10:1 (mL:kg), followed by thoroughly mixing to obtain the experimental feed containing 300 or 1000 mg/kg baohuoside I. The feed was air-dried under ambient conditions and stored at 4 °C.

A total of 240 large yellow croakers (20 ± 5 g) were randomly divided into three groups with 80 fish/tank in a 500 L tank (circulating seawater at 18 ± 2 °C, 25 ± 0.5 ppt, pH 8.0 ± 0.5, dissolved oxygen 7.2 ± 1 mg/L) after acclimatization. Based on preliminary safety assessments, 300 mg/kg and 1000 mg/kg baohuoside I in basal feed administered for 7 days at 3% of body weight were determined to be the maximum safe concentrations. Here, fish were fed with feed containing 300 mg/kg or 1000 mg/kg baohuoside I at 3% of body weight, while the control group received basal feed without baohuoside I, twice daily (10:00 and 22:00) for 28 days. They were challenged with theronts at a concentration of 3000 theronts/fish for 2 h with gentle aeration but without circulating seawater, and then the circulation system was restored to normal culturing for 10 days. During this period, the experimental and control groups were fed with feed containing baohuoside I and basal feed, respectively, and the time to death and number of large yellow croaker in each group were recorded. At 72 h post-infection, five fish from each group were counted for trophonts on bilateral fins and gills under a microscope. The experiments were performed according to the ARRIVE (Animals in Research: Reporting In Vivo Experiments) guidelines [19] and the Guidelines for the Care and Use of Medical Laboratory Animals (Ministry of Health, China, 1998). The procedure was approved and overseen by the Animal Care and Use Ethics Committee of Fujian Agriculture and Forestry University (No. PZCASFAFU22018).

### 2.8. Enzyme Activity Assay

At 72 h post-infection with *C. irritans* theronts, serum was collected from five fish of each group fed with feed containing baohuoside I or basal feed to determine the activities of acid phosphatase (ACP), total superoxide dismutase (T-SOD), and total antioxidant capacity (T-AOC) using the Acid Phosphatase Assay Kit (A060-2-2), Total Superoxide Dismutase Assay Kit (A001-1-2) and Total Antioxidant Capacity Assay Kit (A015-2-1) supplied by Nanjing Jiancheng Bioengineering Institute (Nanjing, China), respectively, according to the manufactures’ instructions.

### 2.9. Transcriptomic Analysis

At 72 h post-infection with *C. irritans* theronts, skin samples (3 cm × 1 cm) were collected from three *C. irritans*-infected fish of each group fed with feed containing baohuoside I at a concentration of 1000 mg/kg or basal feed. Total RNA was extracted from the skin tissues using Trizol Reagent (ER501-01-V2, TransGen, Beijing, China). RNA integrity and contamination were analyzed with 1% agarose gel electrophoresis and the Agilent 4200 system (Agilent Technologies, Waldbronn, Germany). The concentration of the resulting RNA was determined by Qubit 4.0 (Thermo Fisher Scientific, Waltham, MA, USA) and Nanodrop One (Thermo Fisher Scientific, Waltham, MA, USA). The cDNA library was constructed using an ALFA-SEQ RNA Library Prep Kit (NRI001E-03, FINDROP, Guangzhou, China) according to the manufacturer’s instructions. Transcriptome sequencing was performed on the Illumina HiSeq 2000 platform (Illumina, San Diego, CA, USA) to generate 150 bp paired-end reads. Raw data were quality-filtered with fastp (v0.23.2) to obtain clean reads, which were further aligned to the NCBI ribosome RNA database using Bowtie2 (v2.4.5). Clean reads were mapped to the *Larimichthys crocea* reference genome (L_crocea_2.0) using Hisat2 (v2.2.1). Read counts were quantified with RSEM (v1.3.3). Differential expression analysis between groups was performed using DESeq2 (v1.34.0). Genes with a *p*-value < 0.05, false discovery rate (FDR) ≤ 1 and |log2 (Fold Change)| ≥ 1 were defined as differentially expressed genes (DEGs). Functional annotation of DEGs was conducted through Gene Ontology (GO) classification and Kyoto Encyclopedia of Genes and Genomes (KEGG) pathway enrichment analysis using clusterProfiler (v4.2.2). GO terms and KEGG pathways with an FDR ≤ 0.05 were considered significantly enriched.

### 2.10. Quantitative Real-Time PCR (qRT-PCR)

To validate the transcriptomic results, two DEGs (*hsp90aa1.1* and *polr3k*) related to immunity were selected for qRT-PCR analysis. The expression of another two representative genes (*pik3r3* and *ppp3ca*) related to immunity were also analyzed. Total RNA was extracted from the skin samples of three *C. irritans*-infected fish of each group fed with feed containing baohuoside I at a concentration of 1000 mg/kg or basal feed using Trizol Reagent (ER501-01-V2, TransGen, Beijing, China), followed by determination of the concentration and purity with a NanoDrop 2000 Spectrophotometer (Thermo Scientific, Waltham, MA, USA). Synthesis of cDNA was conducted with the PrimeScript RT reagent Kit (RR037A, Kyoto, Japan) according to the manufacturer’s instructions. Real-time PCR was performed using ChamQ Universal SYBR qPCR Master Mix (Q711-03, Vazyme, Nanjing, China) and specific primers for *hsp90aa1.1*, *polr3k*, *pik3r3* and *ppp3ca* as listed in Table 1. The relative expression levels of target genes were normalized to *Lcβ-actin*. Experiments were conducted with three biological replicates, each with three technical replicates.

### 2.11. Hemolysis Assay

The hemolytic activity of baohuoside I was examined in 1% *L. crocea* erythrocytes at concentrations from 12.5 to 800 μg/mL as described previously [20], where erythrocytes treated with 0.9% saline and distilled water were used as a negative (0% hemolysis) and positive control (100% hemolysis), respectively. The supernatant absorbance was measured at 575 nm.

### 2.12. Statistical Analysis

All data were analyzed using the GraphPad Prism 8.0 software (Graphpad, San Diego, CA, USA). Significance of differences was calculated by the one-way ANOVA method followed by the Tukey’s test. Differences with *p* < 0.05 were considered statistically significant. All values are presented as mean values ± standard deviation (SD).

## 3. Results

### 3.1. Chemical Structure Elucidation of Baohuoside I

**Figure 1:**
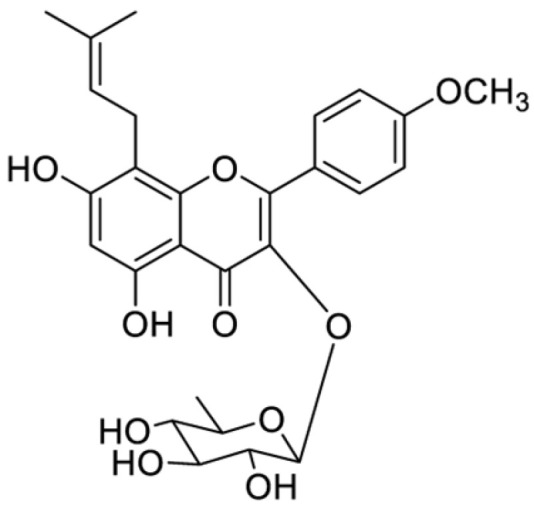
Chemical structure of compound EFrd2 (baohuoside I).

Compound EFrd2 was obtained as yellow powders. ^1^H-NMR (MeOD, 600 MHz) δ ppm: 12.39 (1H, s, HO-5), 7.73 (2H, d, *J* = 8.8 Hz, H-2′/6′), 6.91 (2H, d, *J* = 8.8 Hz, H-3′/5′), 6.17 (1H, s, H-6), 5.08 (1H, t, *J* = 6.6 Hz, H-12), 3.62 (1H, m, Ha-11), 3.36 (1H, m, Hb-11), 3.75 (3H, s, MeO-4′), 1.69 (3H, s, H-14), 1.54 (3H, s, H-15), 5.26 (1H, dd, *J* = 1.2 Hz, H-1″), 3.10–3.68 (H-2″-5″), 0.79 (3H, d, *J* = 5.9 Hz, H-6″). ^13^C-NMR (MeOD, 150 MHz) δ ppm: 154.0 (C-2), 129.6 (C-3), 177.6 (C-4), 161.2 (C-5), 102.9 (C-6), 161.1 (C-7), 107.5 (C-8), 157.0 (C-9), 105.7 (C-10), 24.1 (C-11), 122.7 (C-12), 131.6 (C-13), 27.9 (C-14), 20.3 (C-15), 123.1 (C-1′), 130.7 (C-2′/6′), 158.6 (C-4′), 114.4 (C-3′/5′), 57.0 (MeOD-4′), 99.6 (C-1″), 72.0 (C-2″), 72.1 (C-3″), 72.5 (C-4″), 72.0 (C-5″), 19.5 (C-6″). The spectral data are in good agreement with those reported for baohuoside I [21]. Thus, EFrd2 is a known chemical and identified as baohuoside I. Its molecular formula is C_27_H_30_O_10_ and the structure is shown in Figure 1.

### 3.2. Baohuoside I Exhibited In Vitro Antiparasitic Activity Against C. irritans

**Figure 2:**
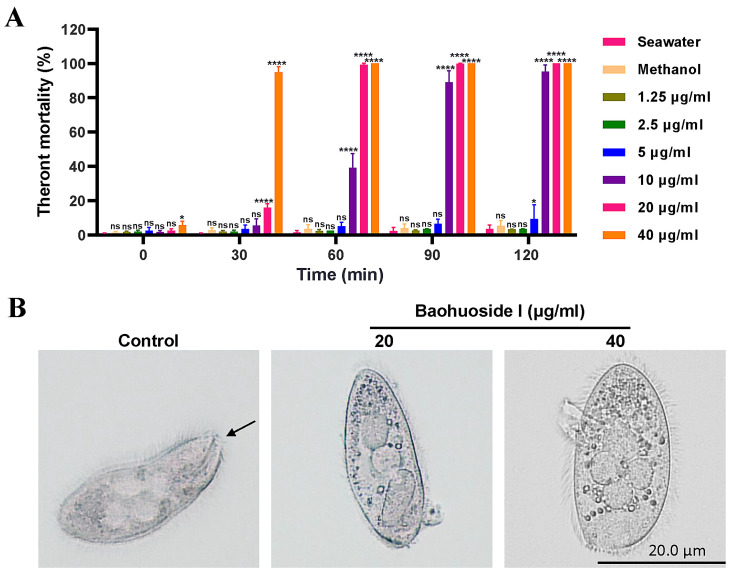
In vitro antiparasitic effects of baohuoside I on *C. irritans* theronts. (**A**) Theront mortality with treatment of baohuoside I for 120 min. ns, no statistical significance, * *p* < 0.05, **** *p* < 0.0001, compared with seawater control. (**B**) Morphology of theronts treated with baohuoside I observed under a microscope (×100 magnification). Control shows normal theronts with intact morphology, visible nucleus, surface cilia, and cytostome (arrow). After treatment with 20 μg/mL baohuoside I for 10 min, cilia detached and vacuolization appeared; after treatment with 40 μg/mL baohuoside I for 30 min, the parasites became rounded, cell membrane ruptured, contents flowed out, and cells lysed.

As shown in Figure 2A, treatment of *C. irritans* theronts with 1.25 μg/mL and 2.5 μg/mL baohuoside for 120 min did not cause detectable mortality. In contrast, exposure to 20 μg/mL and 40 μg/mL baohuoside I for 30 min significantly increased theront mortality. Treated theronts exhibited reduced motility, rounding, and eventual membrane rupture with intracellular contents released (Figure 2B). Theront mortality reached 100% within 60 min, indicating a dose- and time-dependent cytotoxic effect of baohuoside I at concentrations of 20 μg/mL and higher. The IC_50_ values for inhibiting *C. irritans* theronts at 0, 30, 60, 90 and 120 min were 23.33, 21.76, 10.97, 8.03 and 7.54 μg/mL, respectively.

**Figure 3:**
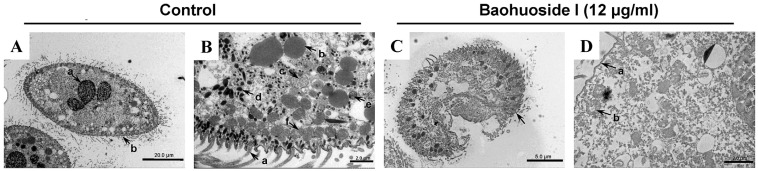
Internal morphology of *C. irritans* theronts observed by transmission electron microscopy. (**A**,**B**) Normal theronts (×700 and ×4000 magnifications). (**A**) Intact parasites, intact nucleus (a) and surrounding cilia (b). (**B**) Clear cilia (a), liposomes (b), Golgi apparatus (c, e), mucous sacs (d), and mitochondria neatly arranged along the inner membrane (f). (**C**,**D**) Theronts treated with 12 μg/mL baohuoside I for 30 min (×2500 and ×5000 magnifications). They show parasite rupture, cilia detachment (arrow in (**C**) and (**D**)-(a)), scattered and damaged mitochondria (b), and disrupted internal structure with no intact organelles visible.

TEM was employed to examine the ultrastructural effects of baohuoside I on *C. irritans* theronts. Figure 3A,B show intact normal theronts with properly organized surface cilia, nuclei, liposomes, Golgi apparatus, mucous sacs, and a single row of mitochondria aligned along the inner membrane; all organelles appear preserved. In contrast, Figure 3C,D display theronts treated with baohuoside I, with detached cilia, dispersed mitochondria, and disrupted organelles, indicating that baohuoside I could compromise the inner membrane-associated mitochondria and various organelles, thereby disrupting theront ultrastructure.

**Figure 4:**
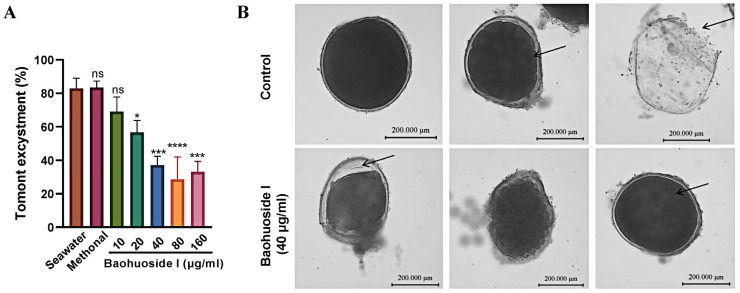
In vitro antiparasitic effects of baohuoside I on *C. irritans* tomonts. (**A**) Tomont excystment with treatment of baohuoside I for 6 h. ns, no statistical significance, * *p* < 0.05, *** *p* < 0.001, **** *p* < 0.0001, compared with seawater control. (**B**) Morphology of tomonts treated with baohuoside I observed under a microscope (×40 magnification). Control shows the normal hatching process of *C. irritans* tomonts, with internal theronts and their emergence from the wall (arrow). After treatment with 40 μg/mL baohuoside I, wall separation was visible, and theronts inside died and could not break out of the wall (arrow).

To assess the impact on tomonts, baohuoside I was applied for 6 h. The hatching rate of tomonts treated with 20 μg/mL baohuoside I was significantly lower than that of the seawater control group, and the hatching rate declined to 37.1% at 40 μg/mL (Figure 4A). Wall separation occurred and the enclosed theronts died and could not break out (Figure 4B), indicating that baohuoside I markedly inhibited tomont excystment at concentrations of 20 μg/mL and higher, with an IC_50_ of 24.98 μg/mL for the inhibition of excystment.

**Figure 5:**
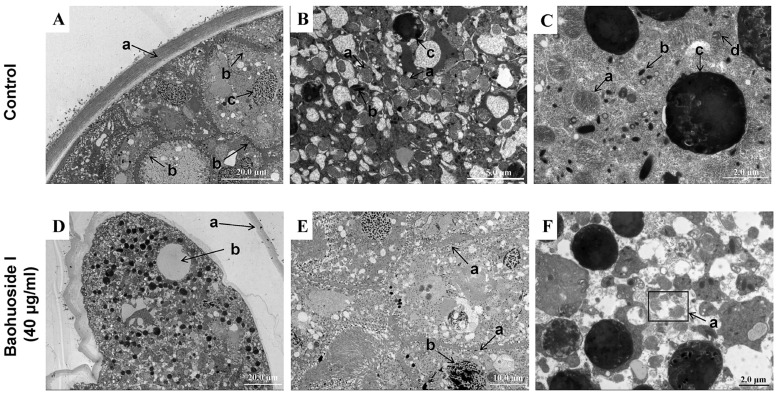
Internal morphology of *C. irritans* tomonts observed by transmission electron microscopy. (**A**–**C**) Normal tomonts (×700, ×3000 and ×6000 magnifications). The wall is intact and tightly attached to the tomont exterior ((**A**)-(a)). Inside are theronts about to hatch and a row of mitochondria neatly arranged beneath the inner membrane ((**A**)-(b)). Theront nucleus is intact ((**A**)-(c)), mitochondria ((**B**)-(a), (**C**)-(a)), mucous sacs ((**B**)-(b), (**C**)-(b)), food vacuoles ((**B**)-(c), (**C**)-(c)), and Golgi apparatus ((**C**)-(d)) are visible, and the cytoplasm is uniform and full. (**D**–**F**) Tomonts treated with 40 μg/mL baohuoside I for 6 h (×500, ×1000 and ×4000 magnifications). Cyst wall separation ((**D**)-(a)), partial wall shrinkage, and vacuolization ((**D**)-(b)) are visible. The structure of mitochondria beneath the inner membrane of some differentiated theronts is destroyed ((**E**)-(a)). Theront nucleus is condensed ((**E**)-(b)), cytoplasm is dispersed with marked vacuolization, and mitochondria are destroyed ((**F**)-(a)).

In TEM observations, normal tomonts were surrounded by an intact wall with theronts about to hatch. Mitochondria were neatly arranged on the inner side of the theront membrane. Nuclei were intact, and organelles including mitochondria, mucous sacs, food vacuoles, and Golgi apparatus were clearly visible with a uniform cytoplasm (Figure 5A–C). After treatment with 40 μg/mL baohuoside I for 6 h, tomonts displayed wall separation and partial shrinkage, accompanied by vacuolization; mitochondria were damaged, nuclei condensed, cytoplasm dispersed, and vacuolization occurred in the enclosed theronts (Figure 5D–F). These ultrastructural changes demonstrated that baohuoside I could induce substantial damage to *C. irritans* tomonts, compromising the integrity of the wall and intracellular organelles.

### 3.3. Effects of Baohuoside I on the Apoptosis of C. irritans

**Figure 6:**
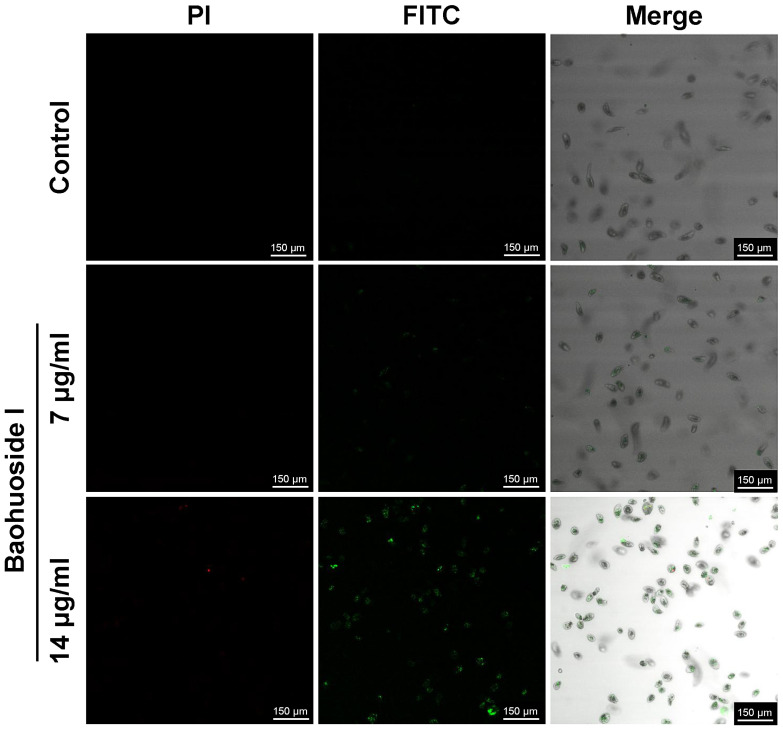
Effects of baohuoside I on the apoptosis of *C. irritans* theronts after a 30 min treatment observed by confocal microscopy (×40 magnification).

**Figure 7:**
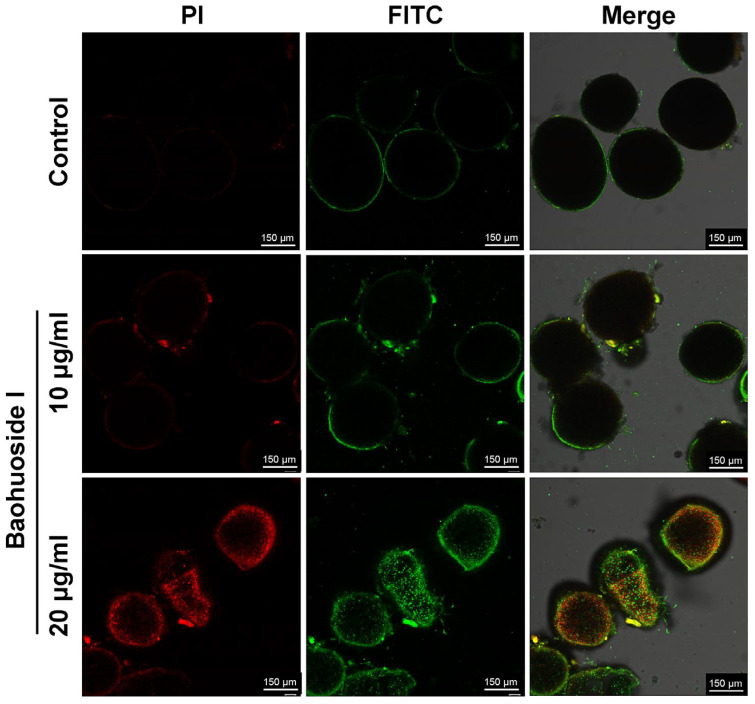
Effects of baohuoside I on the apoptosis of *C. irritans* tomonts after a 12 h treatment observed by confocal microscopy (×40 magnification).

After treatment with 14 μg/mL baohuoside I for 30 min, a large number of *C. irritans* theronts produced green fluorescence, whereas only a few showed red fluorescence. It indicated that phosphatidylserine (PS) on the inner leaflet of the theront membrane had flipped to the outer leaflet, exposing PS to the external environment. Annexin V-FITC bound to the exposed PS, producing green fluorescence. Membrane integrity was preserved in most theronts, as only a minority displayed PI uptake with red nuclear staining, consistent with early apoptosis (Figure 6). After treatment with 20 μg/mL baohuoside I for 12 h, tomonts produced both strong green and red fluorescence (Figure 7), indicating a loss of cell membrane integrity and progression to late apoptosis or even necrosis. Green fluorescence reflects Annexin V-FITC binding to externalized PS, while red fluorescence results from PI uptake and DNA staining in compromised cells.

### 3.4. Effects of Baohuoside I on the Intracellular Ca^2+^ Levels of Tomonts

**Figure 8:**
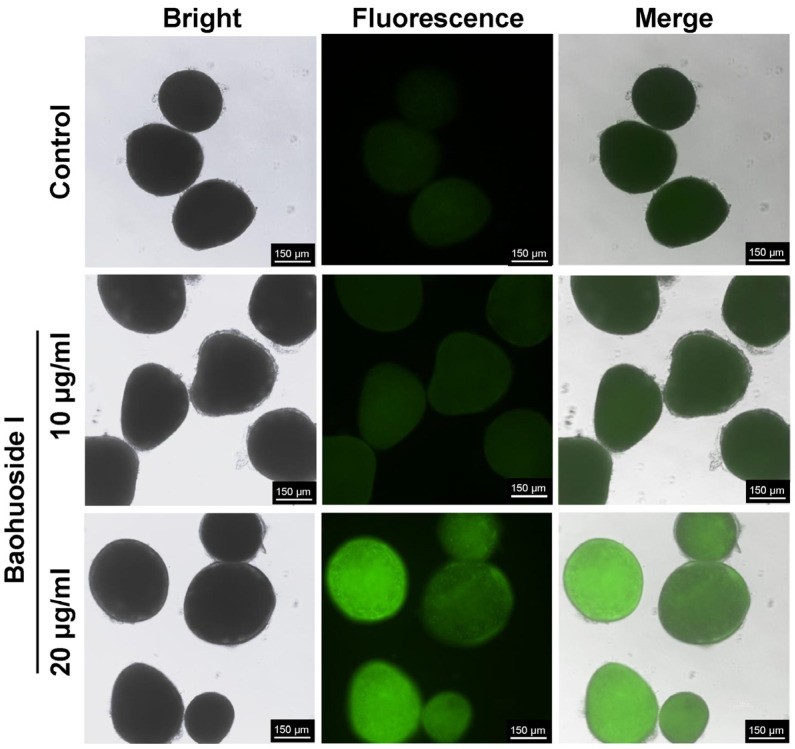
Effects of baohuoside I on the cytosolic Ca^2+^ levels in *C. irritans* tomonts after a 12 h treatment observed under a fluorescence microscope (×40 magnification).

Compared with the control, the fluorescence intensity of tomonts treated with 10 and 20 μg/mL baohuoside I for 12 h was significantly increased in a dose-dependent manner, indicating elevated intracellular Ca^2+^ levels (Figure 8).

### 3.5. Effects of Baohuoside I on the Oxidative Stress of Tomonts

**Figure 9:**
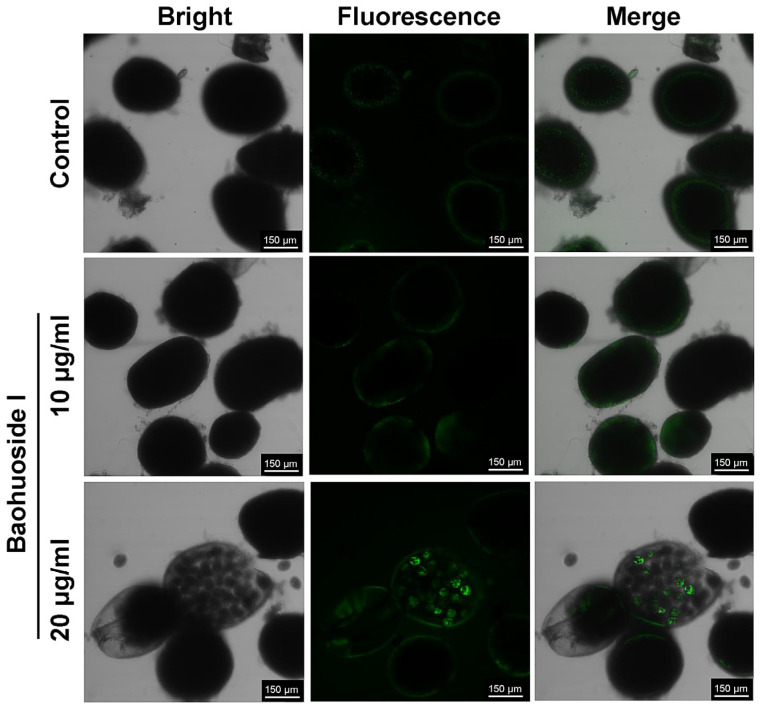
Effects of baohuoside I on ROS levels in *C. irritans* tomonts after a 12 h treatment observed under a confocal microscope (×40 magnification).

Compared with the control, tomonts treated with baohuoside I for 12 h exhibited a significant increase in intracellular fluorescence intensity, indicating elevated ROS levels (Figure 9).

### 3.6. Baohuoside I Exhibited In Vivo Antiparasitic Effects on Fish Challenged with C. irritans

**Figure 10:**
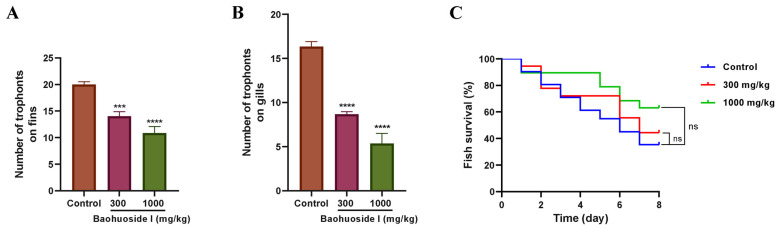
Effects of baohuoside I-treated feed on the prevention of cryptocaryoniasis in large yellow croakers. Number of trophonts on bilateral fins (**A**) and gill arches (**B**) of large yellow croakers at 72 h post-infection with *C. irritans*. Fish were fed with baohuoside I-containing or basal feed for 28 days, then challenged with a sublethal dose (3000 theronts/fish) of *C. irritans* theronts for 2 h, and the number of trophonts on bilateral fins and gill arches was counted at 72 h post-infection. (**C**) Survival rate of large yellow croaker challenged with *C. irritans*. Fish were fed with baohuoside I-containing or basal feed for 28 days and then infected as described and monitored daily for 8 days. ns, no statistical significance, *** *p* < 0.001, **** *p* < 0.0001, compared with control. Control, infected large yellow croakers fed with basal feed.

Before infection with *C. irritans* theronts, large yellow croakers were fed with feed containing baohuoside I or basal feed, and the number of trophonts on bilateral fins and gill arches was analyzed at 72 h post-infection. Compared with the basal feed group, the number of trophonts on the bilateral fins and gill arches decreased by 30% and 46.9%, respectively, in the 300 mg/kg baohuoside I group, and by 45.8% and 67.3%, respectively, in the 1000 mg/kg baohuoside I group (Figure 10A,B). It indicated that baohuoside I could reduce trophont attachment to the body surface and enhance resistance to *C. irritans* infection.

Concurrently, the effects of oral administration of baohuoside I on fish survival were observed for 8 days post-infection. On day 8, the survival rates of large yellow croakers fed with 300 and 1000 mg/kg baohuoside I-treated feed were 44.4% and 63.16%, respectively, both higher than the basal feed control group (35.5%), but differences were not statistically significant (Figure 10C). It illustrated that dietary baohuoside I might improve host resistance to *C. irritans* infection and enhance survival to a certain extent.

### 3.7. Effects of Baohuoside I on Host Defense

**Figure 11:**
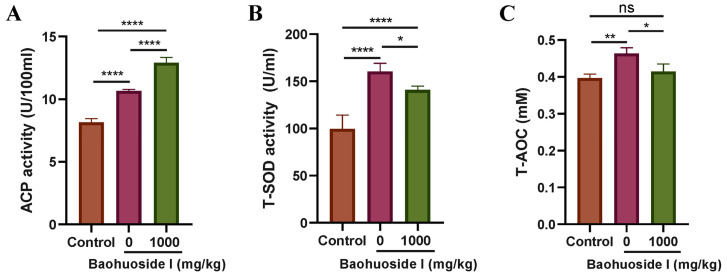
Effects of baohuoside I on serum ACP activity, SOD activity and T-AOC in large yellow croakers. After 28 days of feeding with baohuoside I-containing or basal feed, fish were infected with a sublethal dose (3000 theronts per fish) for 2 h, and serum ACP activity (**A**), SOD activity (**B**) and T-AOC (**C**) were determined at 72 h post-infection. ns, no statistical significance, * *p* < 0.05, ** *p* < 0.01, **** *p* < 0.0001. Control, uninfected large yellow croakers fed with basal feed.

Before infection with *C. irritans* theronts, large yellow croakers were fed with feed containing baohuoside I or basal feed for 28 days. At 72 h post-infection, serum activities of acid phosphatase (ACP) and total superoxide dismutase (T-SOD), as well as total antioxidant capacity (T-AOC), were determined. As shown in Figure 11, infection with theronts significantly increased serum ACP activity, T-SOD activity, and T-AOC in fish fed with the basal diet. Fish fed with baohuoside I exhibited significantly higher ACP activity than the infected basal diet group (Figure 11A), suggesting that baohuoside I enhanced resistance to *C. irritans* by upregulating ACP activity. Serum T-SOD activity in the baohuoside I group was significantly lower than that in the infected basal diet group, and T-AOC levels were reduced to those observed in healthy fish (Figure 11B,C), indicating that baohuoside I alleviated *C. irritans* infection-induced oxidative stress.

### 3.8. Effects of Baohuoside I on the Transcriptome of Fish Responses to C. irritans Infection

To investigate the effects of baohuoside I on the transcriptome of *L. crocea*, skin samples from *C. irritans*-infected fish and baohuoside I (1000 mg/kg)-treated fish (three biological replicates per treatment, six libraries total) were subjected to RNA sequencing. The Illumina HiSeq 2000 run generated a total of 294,231,696 raw reads. After quality filtering, an average of >93.80% of reads per sample were retained as high-quality clean reads. An average of 95.91% of clean reads were mapped to the *L. crocea* genome. The average Q30 quality score was 96.99%, and the average GC content of the reads was 50.02%. These metrics demonstrated that the sequencing data were of high quality and were reliable for the downstream analyses (Table 2). The whole-sequence metadata have been deposited in the NCBI Sequence Read Archive (SRA) database with the accession number PRJNA1405905.

**Figure 12:**
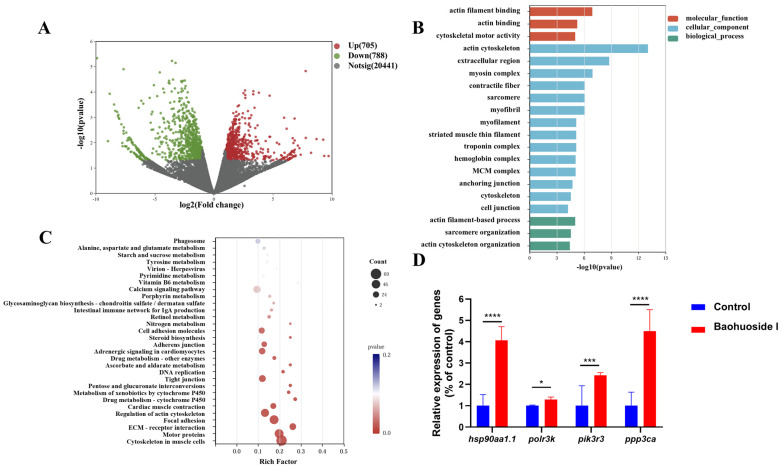
Transcriptome analysis of *C. irritans*-infected *L. crocea*. skin. (**A**) Volcano plots of DEGs. Each point represents a gene; red indicates significantly upregulated DEGs and green indicates significantly downregulated DEGs (FDR ≤ 1, *p*-value < 0.05 and |log2 (Fold Change)| ≥ 1). (**B**) GO enrichment of DEGs showing top GO terms in molecular function (MF), cellular component (CC), and biological process (BP). (**C**) Top 30 enriched KEGG pathways of DEGs. The *x*-axis denotes the Rich Factor (ratio of DEG count to total gene count in each pathway); point size reflects the number of DEGs in that pathway; and point color indicates statistical significance (adjusted *p*-value). (**D**) Expression of *hsp90aa1.1*, *polr3k*, *pik3r3* and *ppp3ca* analyzed by qRT-PCR. *Lcβ-actin* was used as the reference gene. * *p* < 0.05, *** *p* < 0.001, **** *p* < 0.0001. Control, infected large yellow croakers fed with basal feed.

With the thresholds of FDR ≤ 1, *p*-value < 0.05 and |log2 (Fold Change)| ≥ 1, a total of 1493 genes were identified as differentially expressed in the skin of baohuoside I-treated *L. crocea* compared with the untreated controls, including 705 significantly upregulated and 788 downregulated genes (Figure 12A and Table S1). GO enrichment analysis showed that the DEGs were significantly enriched in 3 terms of the molecular function (MF), 14 terms of the cellular component (CC), and 3 terms of the biological processes (BP) (Figure 12B and Table S2). KEGG enrichment analysis indicated that DEGs were predominantly enriched in pathways related to cell motility, and signaling molecules and interaction, including cytoskeleton in muscle cells, motor proteins, and ECM–receptor interaction (Figure 12C and Table S3).

Based on the results of GO and KEGG analysis, a large number of immune-related genes were significantly upregulated, such as heat shock protein HSP 90-alpha 1 (*hsp90aa1.1*), RNA polymerase III subunit K (*polr3k*), purinergic receptor P2X 7 (*p2rx7*), and toll like receptor 3 (*tlr3*). Conversely, the expression levels of genes such as SAM and HD domain containing deoxynucleoside triphosphate triphosphohydrolase 1 (*samhd1*), TBK1-binding protein 1 (*tbkbp1*), phospholipase C beta 3 (*plcb3*), phospholipase C beta 2 (*plcb2*), and C-C motif chemokine receptor 6 (*ccr6*) were significantly downregulated. *Hsp90aa1.1* and *polr3k* were selected for qPCR validation. Compared with the *C. irritans*-infected fish fed with basal feed, both genes were significantly upregulated in the skin of baohuoside I-treated fish, consistent with the transcriptomic results. Additionally, the expression levels of immune-related genes phosphatidylinositol 3-kinase regulatory subunit gamma (*pik3r3*) and protein phosphatase 3 catalytic subunit alpha (*ppp3ca*) were also significantly upregulated in the baohuoside I group (Figure 12D).

### 3.9. Hemolytic Activity of Baohuoside I

**Figure 13:**
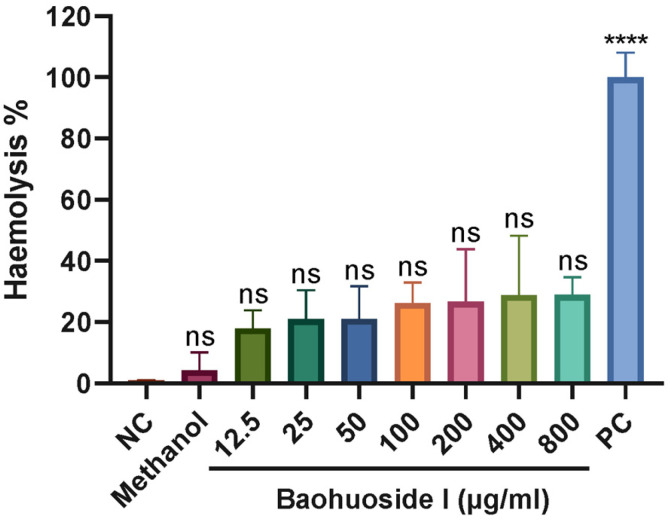
Hemolytic effect of baohuoside I on large yellow croaker erythrocytes. Baohuoside I was incubated with fish erythrocytes for 60 min, and the supernatant absorbance was measured at 575 nm. ns, no significant difference, **** *p* < 0.0001, compared with negative control. Negative control (NC), erythrocytes incubated with 0.9% saline; positive control (PC), erythrocytes incubated with distilled water.

To evaluate the safety of baohuoside I, erythrocytes from large yellow croakers were co-incubated with baohuoside I. It displayed that 12.5–800 μg/mL baohuoside I did not induce hemolysis (Figure 13), indicating no hemolytic activity at the concentrations effective for inhibiting tomont hatching and killing theronts.

## 4. Discussion

Cryptocaryoniasis is one of the principal threats to marine cultured fish in tropical and subtropical regions. Traditional control strategies, including physical methods, chemical treatments, and vaccination, pose risks to fish health, product safety, and the ecological environment, and their practical effectiveness and cost-effectiveness are often limited. Therefore, effective, economical, and safe approaches to control *C. irritans* are still needed. This study did not include positive controls (e.g., copper sulfate, hyposalinity, and temperature adjustment) as it mainly aimed to evaluate the antiparasitic activity and mechanisms of baohuoside I rather than comparing the efficacy with conventional treatments that differ substantially in mode of action.

The theront stage of *C. irritans* is a critical period for preventing white spot disease. In recent years, screening substances with anti-*C. irritans* activity from natural products has emerged as a promising strategy for the control of cryptocaryoniasis. Among the tested phytochemicals, baohuoside I exhibited superior anti-*C. irritans* activity. For example, matrine, oxymatrine, caprylic acid, epigallocatechin gallate (EGCG), and 3,4-dihydroxy-L-phenylalanine (L-DOPA) required 402.6 μM, 378 μM, 500 μM, 109 μM and 507 μM, respectively, to achieve 100% theronts mortality within 2–3 h [22,23,24], whereas 40 μg/mL (78 μM) baohuoside I achieved 100% theront mortality within 60 min. In vivo evidence further supports the potential of baohuoside I. *Pagrus major* fed with 0.5 g/kg matrine- or oxymatrine-treated feed for 7 days, followed by a 3-day feeding after *C. irritans* infection, showed reductions in gill trophonts of 58% and 32%, respectively [22]. In the present study, large yellow croaker were fed with 1 g/kg baohuoside I-treated feed for 28 days before infection and for 2 days afterward, resulting in a trophont reduction of 67.3% on the gills. However, the anti-*C. irritans* effect of baohuoside I was not as good as that of honokiol, which induced 100% theront death within 30 min at 1.0 μg/mL and achieved a reduction of 91% in gill trophonts in pompano (*Trachinotus ovatus*) fed with 0.4 μg/kg for 7 days and then 3 days post-infection [25]. TEM revealed that baohuoside I induced apoptosis-like morphology in theronts and tomonts, aligning with the apoptosis-like death induced by other anti-protozoal agents such as those against *Leishmania* sp. and *Ichthyophthirius multifiliis* [26,27]. In the hemolysis assay, 800 μg/mL baohuoside I, approximately 20-fold the concentration required to inhibit tomont hatching and kill theronts, did not cause hemolysis of large yellow croaker erythrocytes, indicating a favorable safety margin for erythrocytes.

Dysregulation of intracellular Ca^2+^ homeostasis serves as a pivotal early event triggering apoptosis. After a 12 h treatment with baohuoside I at 10 and 20 μg/mL, tomonts exhibited a dose-dependent rise in intracellular Ca^2+^ levels (Figure 8). Ca^2+^ overload could initiate the intrinsic apoptotic pathway via activation of calcium-dependent proteases (e.g., calpains) and mitochondrial dysfunction [28,29]. In *C. irritans* tomonts, honokiol elevated the intracellular Ca^2+^, leading to mitochondrial membrane potential decline, cytochrome c release, and caspase cascade activation [30]. Similarly, the present study’s TEM observations of mitochondria damage and nuclear condensation in theronts and tomonts were consistent with the Ca^2+^-mediated apoptotic pathways. Notably, transcriptomic analysis further showed enrichment of DEGs in the “Calcium signaling pathway”, with notable upregulation of Ca^2+^-related genes in fish skin, such as *pik3r3* and *ppp3ca*, indicating that baohuoside I could modulate host Ca^2+^ homeostasis to aid resistance against parasitism; however, the precise mechanisms by which it regulated host skin Ca^2+^ homeostasis need further investigation.

Flavonoids are well-known for their dual antioxidant/prooxidant properties, and their antiparasitic effects are frequently attributed to prooxidant mechanisms [31,32]. ROS played a dual role in baohuoside I-induced cell death. Treatment of baohuoside I markedly increased ROS levels in tomonts (Figure 9), consistent with the ROS-driven apoptotic mechanisms described for other natural products, such as 4′,7-dihydroxyflavone against *Leishmania* sp. [26]. Excess ROS can trigger lipid peroxidation, protein oxidation, and DNA damage, compromising organelles and cell membranes [33,34]. The observed vacuolization, mitochondria disintegration, and shrinkage and separation of the tomont wall (Figure 5) are characteristic of ROS-mediated oxidative damage. Concurrently, ROS forms a positive feedback loop with Ca^2+^ overload. Ca^2+^-induced dysfunction in the mitochondrial electron transport chain (ETC) leads to electron leakage and the generation of more ROS, which in turn further impairs Ca^2+^ pumps and membrane integrity, amplifying Ca^2+^ influx [35]. Such a self-reinforcing toxic cycle ultimately resulted in the death of theronts and inhibition of tomont excystment. Moreover, protozoa typically possess limited antioxidant defense systems, reducing their capacity to neutralize reactive oxygen and nitrogen species (ROS/RNS), rendering them particularly susceptible to prooxidant compounds [36]. The observed apoptosis-like death in both theronts and tomonts (Figure 6 and Figure 7), characterized by phosphatidylserine externalization and membrane permeabilization, is likely mediated by ROS-dependent activation of the intrinsic apoptotic pathway [37]. While this study did not directly measure key apoptotic regulators such as the BAX/Bcl-2 ratio or caspase-3/9 activation to fully characterize the downstream signaling cascade, the observed mitochondrial dysfunction and ROS accumulation strongly suggested the activation of mitochondrial-dependent apoptotic pathways [38].

Natural products with antiparasitic mechanisms similar to baohuoside I include honokiol and magnolol. Zhao et al. reported that treating *C. irritans* tomonts with 1 μg/mL honokiol caused a rapid rise in intracellular Ca^2+^, followed by increases in ROS levels, caspase-3/9 activity, and DNA fragmentation at 2 h, along with a decrease in mitochondrial membrane potential; the apoptosis-like mortality peaked at 4 h [30]. Furthermore, magnolol and honokiol have been shown to inhibit ETC, obstruct mitochondrial energy metabolism and affect ATP-binding cassette (ABC) transporters, thereby weakening detoxification and xenobiotic excretion in worms [39]. Compared with honokiol, baohuoside I required a relatively higher effective concentration (about 20 μg/mL) and a longer exposure (30 min for theronts, 6 h for tomonts). It might be attributed to the differences in compound structure, target affinity, and cellular uptake efficiency. Notably, the effects of baohuoside I on modulating host skin Ca^2+^ signaling pathways, such as the upregulation of *pik3r3* and *ppp3ca* genes, may offer a dual advantage by disrupting parasite Ca^2+^ homeostasis while enhancing host immune signaling.

In the in vivo challenge experiment, ACP activity, T-SOD activity and T-AOC in the serum of the infected fish were significantly elevated (Figure 11), reflecting that infection induced pronounced oxidative stress and immune activation. The serum ACP activity in baohuoside I-fed fish was significantly higher than that in the infected control group, suggesting that baohuoside I might promote the clearance of attached or invading *C. irritans* trophonts by enhancing the phagocytic and killing capabilities of immune cells such as macrophages and neutrophils [40,41]. Transcriptomic analysis showed significant enrichment of DEGs in the “Phagosome” pathway, alongside upregulation of immune-related genes such as *hsp90aa1.1*, *polr3k*, *p2rx7*, and *tlr3*, evidencing the enhancement of phagocytic function by baohuoside I. Conversely, serum T-SOD activity and T-AOC in baohuoside I-fed fish were significantly lower than those in the infected control group, indicating that baohuoside I might attenuate infection-induced oxidative stress by effectively eliminating parasites, reducing ROS accumulation in host cells, and upregulating endogenous antioxidant genes. The enrichment of the “Intestinal immune network for IgA production” pathway also suggested that baohuoside I might improve mucosal barriers to reduce pathogen invasion, alleviating oxidative stress. This bidirectional regulation, prooxidant in parasites and antioxidant in hosts, highlights the potential of baohuoside I as a selective therapeutic agent with minimal collateral damage to host tissues [42].

The regulatory effects of baohuoside I on the host immunity and antioxidant systems confer advantages relative to other natural products. For example, vitamin C primarily enhanced fish immunity by boosting non-specific defense enzymes [41], whereas baohuoside I not only enhanced ACP-mediated phagocytosis but also alleviated oxidative stress, potentially avoiding excessive inflammatory and oxidative damage associated with broad immune activation. Compared with honokiol, baohuoside I appeared to exert a stronger immunomodulatory effect on the host. Honokiol could directly induce parasite apoptosis, yet studies on its regulation in host immune system are limited. In contrast, baohuoside I activated multiple immune-related pathways, including phagosomes, calcium signaling pathways, and intestinal immune networks, and significantly upregulated the expression of *hsp90aa1.1* and *polr3k*, demonstrating a more comprehensive immunomodulatory and tissue-protective function.

Transcriptomic analysis provided a comprehensive view of baohuoside I-mediated antiparasitic mechanisms against *C. irritans*. GO enrichment showed that DEGs were significantly enriched in “actin filament-based process”, “actin cytoskeleton organization”, and “sarcomere organization” categories, implying that baohuoside I might promote cytoskeletal rearrangement in immune cells to enhance migration and phagocytic capacity for eliminating *C. irritans* trophonts more effectively. It aligns with the reports that macrophage and neutrophil phagocytosis in fish relies on dynamic actin remodeling [43,44]. The observed elevation of serum ACP activity further supports enhanced phagocytic function.

KEGG enrichment further revealed the multi-target mechanism of baohuoside I. Among the top 30 enriched KEGG pathways, “Phagosome” matched the elevated serum ACP activity and cytoskeletal reorganization in GO analysis, further supporting the effect of baohuoside I on enhancing the host phagocytic response. Upregulation of *hsp90aa1.1* and *polr3k* in the skin of baohuoside I-fed fish also indicated immune enhancement, as HSP90 participates in antigen presentation, pro-inflammatory cytokine production, and cell survival regulation [45], and *polr3k* is associated with the transcriptional regulation of immune genes [46].

The enrichment in “Intestinal immune network for IgA production” suggested the reinforcement of mucosal barriers, with the upregulation of *tlr3* further indicating enhanced pattern recognition receptor-mediated innate immune responses [47]. The upregulation of *p2rx7* indicated the purinergic signaling involvement in inflammasome activation and cytokine release [48]. In contrast, the significant downregulation of *samhd1*, *tbkbp1*, *plcb3*, *plcb2* and *ccr6* reflected the suppression of excessive inflammatory responses to prevent immunopathology, suggesting that baohuoside I finely modulated immune responses to balance antiparasitic activity and inflammation.

The enrichment in the “Calcium signaling pathway”, together with the significant upregulation of *pik3r3* and *ppp3ca*, indicated that baohuoside I modulated Ca^2+^ signaling networks in the host [49]. PI3K and calcineurin pathways are central to T cell activation and immunomodulation [49,50,51]; therefore, upregulation of *pik3r3* and *ppp3ca* implied enhanced host immune cell functions via optimized Ca^2+^ signaling. This dual action, disrupting parasite Ca^2+^ homeostasis while augmenting host Ca^2+^-dependent signaling, may contribute to the high efficacy and safety of baohuoside I.

Enrichment of pathways related to cell junctions and extracellular matrix, including “Tight junction”, “Adherens junction”, “Focal adhesion” and “ECM–receptor interaction”, suggested promotion of tissue repair and barrier restoration, which could mitigate *C. irritans*-induced tissue damage and improve host survival. Compared with compounds with solely direct antiparasitic effects, baohuoside I may accelerate recovery from infection by facilitating barrier repair and immune regulation.

Overall, baohuoside I exhibited a distinctive mode of action. For instance, honokiol primarily induced endoplasmic reticulum stress and disrupted calcium homeostasis [30]; whereas baohuoside I not only affected calcium signaling pathways in tomonts but also combined host immune activation, phagocytic enhancement, Ca^2+^ signaling modulation, and tissue repair processes to achieve antiparasitic efficacy with host protection. Moreover, the regulation of actin cytoskeleton and muscle-related genes by baohuoside I was relatively special, suggesting its ability to enhance immune cell migration and phagocytic function. The robust upregulation of key immune genes (*hsp90aa1.1*, *polr3k*, *p2rx7*, *tlr3*, *pik3r3* and *ppp3ca*) and the coordinated downregulation of inflammatory regulators (*samhd1*, *tbkbp1*, *plcb3*, *plcb2* and *ccr6*) suggested a balanced immune response that supported parasite clearance while limiting immunopathology. This bidirectional regulation of Ca^2+^ signaling, as well as the integrated modulation in immune response and activation of phagosomal and barrier-associated pathways may underlie the strong in vivo efficacy and safety of baohuoside I against *C. irritans*. Although some other flavonoids such as luteolin, quercetin and baicalein also exhibited antiparasitic effects against various protozoan parasites including *Leishmania donovani* and *Trypanosoma cruzi* [52,53], baohuoside I represents the first *Epimedium*-derived flavonoid with antiparasitic activity against *C. irritans*, exhibiting distinctive immunomodulatory effects on the host, which have not been reported for other flavonoids.

Although baohuoside I demonstrated promising antiparasitic efficacy, there are some limitations to this study. (1) The withdrawal time for baohuoside I in *L. crocea* has not been established. (2) The in vivo trials were conducted under controlled laboratory conditions, which might not fully reflect the complex environmental factors in real mariculture settings. (3) The economic feasibility and long-term effects of baohuoside I require further evaluation. Although *E. brevicornu* is abundant in China and baohuoside I constitutes 0.017–0.197% of its dry weight [54,55], large-scale production costs need optimization to compete with inexpensive chemical agents like copper sulfate. Biotechnological approaches may offer cost-effective alternatives in the future. Regarding long-term effects, baohuoside I showed favorable preliminary safety profiles (no hemolysis at 800 μg/mL, no acute mortality) consistent with the traditional use of *E. brevicornu*. However, chronic toxicity, resistance development, and environmental risks require systematic evaluation. (4) This study did not validate ROS-dependent effects using antioxidant scavengers (e.g., N-acetylcysteine, glutathione) or directly measure ROS/MAPK signaling pathway components (e.g., BAX/Bcl-2 ratio, caspase-3/9 activation) to fully characterize the downstream apoptotic cascade [56].

Future work would include systematic pharmacokinetic studies and residue monitoring for the determination of the appropriate withdrawal period for baohuoside I in aquaculture applications, field trials with larger sample sizes under actual farming conditions for the validation of the practical efficacy and optimal application protocols of baohuoside I, optimizing extraction processes to reduce production costs, comprehensive assessments of economic feasibility, chronic toxicity, ecotoxicity, resistance development, and environmental risks, as well as exploration of ROS/MAPK-mediated mechanisms and specific redox-sensitive targets of baohuoside I in *C. irritans*.

## 5. Conclusions

This study is the first to reveal that baohuoside I exhibits anti-cryptocaryoniasis efficacy through a multifaceted and multitargeted mechanism. It directly induces parasite apoptosis and disrupts intracellular Ca^2+^ and ROS homeostasis. Simultaneously, baohuoside I improves host defenses by enhancing innate immunity, phagocytosis, tissue repair, and Ca^2+^ signaling modulation, as well as mitigating oxidative stress. This synergistic action, combining direct antiparasitic effects with host protection, makes baohuoside I a promising therapeutic candidate for cryptocaryoniasis with significant aquaculture potential.

## Figures and Tables

**Table 1:** Primers used for qRT-PCR.

Genes	Forward (5′-3′)	Reverse (5′-3′)	Gene Accession Number
*hsp90aa1.1*	GTACCCAGAAGGGCTGCATT	GTTGTCCTTGTCCTCTGCGA	XM_010738125
*polr3k*	CTGCAGCTTGGGAAAACGTG	GGTGTCCACATTGGGCGTTA	XM_010730533
*pik3r3*	AGTCAAAGGGGGAAGTACGG	GTCAACTTCCATCACAACCTGC	XM_010742691
*ppp3ca*	AGCCATTGAAGCCATCGAGC	TTGTCATCCGTGCCGTTTGC	XM_010738001
*Lcβ-actin*	GACCTGACAGACTACCTCATG	AGTTGAAGGTGGTCTCGTGGA	XM_027284923

**Table 2:** The quality of transcriptome sequencing data mapping to the reference genome of *L. crocea*.

Sample	Raw Reads	Clean Reads (% of Raw)	Total Mapped Reads (% of Clean)	Q30 (%)	GC Content (%)
*Lc*S1	52,354,504	48,621,152 (92.87)	46,158,636 (94.94)	96.56	50.48
*Lc*S2	49,416,800	45,728,766 (92.54)	43,391,941 (94.89)	96.64	49.49
*Lc*S3	48,139,934	46,882,664 (97.39)	45,920,732 (97.95)	97.88	50.92
*Lc*ST1	56,404,654	54,035,104 (95.80)	53,168,200 (98.40)	97.85	50.82
*Lc*ST2	43,447,204	39,959,548 (91.97)	37,857,599 (94.74)	96.57	48.22
*Lc*ST3	44,468,600	41,015,042 (92.23)	38,761,499 (94.51)	96.44	50.20

*Lc*S: skin of infected *L. crocea*. *Lc*ST: skin of baohuoside I-treated *L. crocea*. Q30: percentage of bases with Phred quality ≥ 30. GC content: percentage of G + C bases.

## Data Availability

The original contributions presented in this study are included in the article and Supplementary Materials. Further inquiries can be directed to the corresponding authors. The original RNA sequence metadata are openly available in the NCBI Sequence Read Archive (SRA) database with the accession number PRJNA1405905 (https://www.ncbi.nlm.nih.gov/sra/PRJNA1405905, accessed on 6 March 2026).

## Supplementary Materials

The following supporting information can be downloaded at https://www.mdpi.com/article/10.3390/antiox15030396/s1. Table S1: Differentially expressed genes (DEGs) in the skin of *C. irritans*-infected *L. crocea* fed with feed containing baohuoside I vs. basal feed. Table S2: GO enrichment of DEGs. Table S3: KEGG enrichment of DEGs.

## Abbreviations

The following abbreviations are used in this manuscript:

TLC	Thin-layer chromatography
TEM	Transmission electron microscope
ROS	Reactive oxygen species
ACP	Acid phosphatase
T-SOD	Total superoxide dismutase
T-AOC	Total antioxidant capacity
DEG	Differentially expressed gene
GO	Gene Ontology
KEGG	Kyoto Encyclopedia of Genes and Genomes
EGCG	Epigallocatechin gallate
L-DOPA	3,4-dihydroxy-L-phenylalanine
ETC	Electron transport chain
ABC	ATP-binding cassette
